# Mapping and Characterizing Instruments for Assessing Family Nurses’ Workload: Scoping Review

**DOI:** 10.3390/nursrep14030151

**Published:** 2024-08-21

**Authors:** António Dias, Beatriz Araújo, Élvio Jesus

**Affiliations:** 1 Unidade de Saúde Familiar Saúde no Futuro, Unidade Local de Saúde Gaia e Espinho, 4400-043 Vila Nova de Gaia, Portugal; 2 Centre for Interdisciplinary Research in Health (CIIS), Universidade Católica Portuguesa, 4169-005 Porto, Portugal; baraujo@ucp.pt (B.A.); ejesus@ucp.pt (É.J.)

**Keywords:** workload, family nursing, scoping review

## Abstract

Background: The importance of knowing the workload of family nurses lies essentially in the possibility of improving health outcomes, care processes and the nurse’s professional life. There is a lack of studies that fully describe the nursing workload in primary care, particularly, in the context of family health nursing, and the ideal metrics to be used remain unknown, making it impossible to characterize and therefore provide the necessary insight to acknowledge the different contributions of several aspects that embody the global workload of family nurses. The objective of this scoping review was to map the known evidence and characterize the instruments used to assess the workload of family nurses. Methods: Scoping review, according to the Joanna Briggs Institute, proposed a methodology for scoping reviews, consisting of three research stages: (1) an initial research in Medline and CINHAL; (2) an extended search, using keywords and search terms, in the following databases: JBI, CINAHL Complete, MEDLINE, Cochrane and Scopus; and (3) a search of the reference lists of the selected articles. No time limit was defined. Results: Fourteen studies referring to ten assessment instruments were included. Nine of them analyze workload as a dimension of a broader instrument, and two studies refer to an instrument that focuses exclusively on workload. Conclusions: The diversity of professional competencies and contexts, the conceptual complexity of workload and the absence of a theoretical framework make it difficult to identify consensual instruments to assess the workload of family nurses. This study was prospectively registered with the Open Science Framework^®^ on 6 September 2023, with the registration number: 3k6vr.

## 1. Introduction

It is essential to assess a nurse’s workload to guarantee high-quality patient care, improve health outcomes and care processes [1], maintain patient safety by reducing adverse events, ethical and legal issues, and costs [2,3], and support nurses’ well-being by preventing professional dissatisfaction, stress, and burnout. Also, health organizations rely on nursing workload assessments to optimize resource allocation, allowing for better staffing and resource management, and ensuring that nursing care processes run efficiently [4].

However, the contributions to the nursing workload are not clearly defined. Different concepts of direct care, such as intensity of care, dependence, the severity of health condition, or complexity of care, related to the nursing workload, are used in similar ways, although they tend to represent different aspects of the essence of care provided by nurses [5]. Furthermore, the scope of the nursing workload is not limited to direct care but also includes other types of care: indirect care and professional development activities or organizational tasks [6]. However, there is a consensus that they all globally influence a nurse’s workload [7].

Also, besides having no ideal method for assessing nursing workload, there has been a rising concern over the emotional aspects of work. This matter should not be overlooked, because its potential usefulness seems to be important in the global assessment of workload [7]. Nurses’ workload is influenced by several emotional aspects of work that can contribute to creating supportive work environments and significantly impact nurses’ performance and well-being, such as emotional exhaustion, regarding a large amount of emotional labor or even compassion fatigue; work-life balance; job satisfaction; and even personality traits, such as resilience and emotional intelligence [8,9,10].

Therefore, challenges persist in the assessment of nurses’ workload, mainly because it is almost impossible to measure the nurses’ entire scope of intervention, which makes it difficult to adopt a single method for determining workload.

Despite this difficulty, the literature suggests that workload assessment can be defined as a method of quantifying the activities, processes and time spent by nurses to provide care [5].

Workload assessment tools should allow researchers to comprehensively identify and evaluate the various variables contributing to workload without compromising feasibility, ultimately enabling the assessment of its impact.

The attempts, described in the literature, to assess nurses’ workload are essentially focused on the hospital environment, particularly in those with high differentiation of care, such as intensive care [11,12]. Furthermore, they focus essentially on direct care and usually include a prior assessment of both the clients’ dependency and the complexity of care associated [4]. Also, emotional aspects are frequently overlooked [8,9,10].

However, for family nurses, these metrics are not appropriate, due to the broad scope of care they provide, which includes promoting health and providing care throughout the life cycle of families and individuals. This span of care contributes largely to the lack of consensus regarding the essential characteristics of workload in primary care and stands as a major difference in the scope of care and professional role provided by family nurses compared to those performed in other professional settings, such as hospitals. Therefore, major differences between professional settings where nurses perform must be acknowledged when choosing workload assessment methods.

The aspects that seem to contribute to a family nurse’s workload remain unclear. Although the literature refers to complex nursing diagnosis, organizational workflows, and the availability of resources as some of the major factors that impact family nurses’ workload, there are still some gaps in the literature, with insufficient data persisting, which makes its assessment difficult [5,13].

The ideal metrics to be used for assessing a family nurse’s workload don’t appear to be thoroughly updated in the instruments traditionally used in hospital settings. Therefore, there is a need to identify instruments and methodologies that more accurately reflect the various aspects of a family nurse’s workload [4]. This means that a comprehensive assessment of workload is a priority and should incorporate the global care provided by family nurses, accommodating, among others, the aspects inherent to both the characteristics of the clients and nurses, the complexity of nursing interventions, the level of demand and the organizational environment [14].

Knowledge of a nurse’s workload, in the specific context of family health nursing, is insufficient, and published studies on this topic are scarce and dispersed in the literature, making it difficult to formulate precise questions. For that reason, we decided to carry out a scoping review, with the objective of mapping the known evidence and characterizing the instruments used to assess the workload of family nurses. Specifically, this scoping review aims to answer the following questions: “What instruments allow the workload assessment of family nurses?” and “What dimensions and variables are present in the workload assessment instruments of family nurses?”

Knowing the answers to these questions will present fundamental contributions to the construction of workload assessment methodologies for family nurses.

## 2. Materials and Methods

This scoping review followed the methodological guidelines proposed by the Joanna Briggs Institute (JBI) [15] and used the Preferred Reporting Items for Systematic Reviews and Meta-Analyses extension for Scoping Reviews (PRISMA-ScR) [16]. It was carried out over five stages: (1) the definition of a research strategy; (2) the identification of relevant studies; (3) study selection; (4) data extraction; and (5) the presentation and discussion of the results.

### 2.1. Search Strategy and Study Identification

We carried out a preliminary search in MEDLINE (via PubMed), the Cochrane Database of Systematic Reviews, the Joanna Briggs Institute, Evidence Synthesis, PROSPERO, and the Open Science Framework, which revealed a lack of literature reviews, published or to be carried out, in this specific problematic.

Therefore, following the JBI methodology, eligibility criteria were defined based on participants, concept, and context (PCC). Regarding participants (P), studies were considered if they focused on family nurses who assume responsibility for providing global nursing care to individuals and families, throughout all stages of the life cycle. Regarding concept (C), we included studies that presented instruments that allowed for the assessment of family nurses’ workload. As for the context (C), studies carried out within the scope of primary health care were included, particularly in the practice environments of family nurses.

Regarding the type of study, we only considered primary and secondary quantitative studies that used objective workload assessment instruments, excluding qualitative or mixed studies that described subjective assessment methodologies, such as interviews. Additionally, the literature reviews, reports, theses, and dissertations, as well as the gray literature, were considered. No time limit was defined as the aim was to encompass the entire corpus of knowledge on this issue.

Regarding the research strategy and identification of studies, the electronic databases CINAHL Complete (via EBSCOhost), MEDLINE (via PubMed), and Scopus were used, given their relevance and as they comprehensively cover the literature on this subject. The search for unpublished studies included the Portuguese Open Access Scientific Repository.

The search strategy aimed to locate published and unpublished studies and was carried out in three stages.

Initially, a conventional search was carried out, limited to the MEDLINE (via PubMed) and CINAHL Complete (via EBSCOhost) databases, to identify the ideal search terms.

Based on these terms, and through the analysis of the words contained in the title, abstract, and keywords used to describe the articles found in the initial search, a complete search strategy was adopted. Using a process of gradual refinement, the aim was to combine the identified keywords and descriptors, adjusted according to the specificities of each database/repository included in the review, using Boolean phrases to carry out the research. Combinations of MeSH descriptors were used using the Boolean operators “OR”, “AND” and the “*” tool, which enhanced the search by creating new variations of the same words. An example is shown in Table 1.

In the third stage, we analyzed the reference lists of all studies selected for critical evaluation to verify the existence of additional eligible studies.

Our search considered keywords in the title and abstract of full-text articles in Portuguese, Spanish, or English, that identified instruments that evaluated the workload of family nurses.

Because this scoping review is a secondary study, for which scientific evidence is made available in the public domain and, therefore, not involving human beings, it was not considered necessary to request an opinion from an ethics committee.

### 2.2. Study Selection

After carrying out the search, all study titles were extracted and stored, using the Rayyan^®^ platform https://rayyan.qcri.org (accessed on 30 June 2022). Duplicate studies were eliminated, and, subsequently, all titles and abstracts were read and analyzed, with the purpose of validating their relevance. Relevant full studies were retrieved, strictly following the review’s inclusion and exclusion criteria. Subsequently, the full text of the studies was evaluated in detail. Regarding the identification of studies from the reference lists, the same procedure was followed.

In case of doubt or disagreement, the full article was retrieved to decide their inclusion. Abstracts and posters published at conferences, as well as opinion articles, were excluded.

As our study is a scoping review, we did not conduct any methodological quality appraisal of the included studies since its aim is to map the existing scientific evidence [15].

### 2.3. Data Extraction

Data extraction was carried out by two reviewers, independently. Any disagreement between the reviewers was resolved through recourse to a third reviewer. The extraction of specific details such as title, authors, country, year of publication, objectives, study design, sample size, characteristics of reported instruments (dimensions, variables, and items), and relevant results was carried out using an extraction instrument, made by the researchers specifically for this scoping review, following the objectives and questions of this review.

## 3. Results

**Figure 1:**
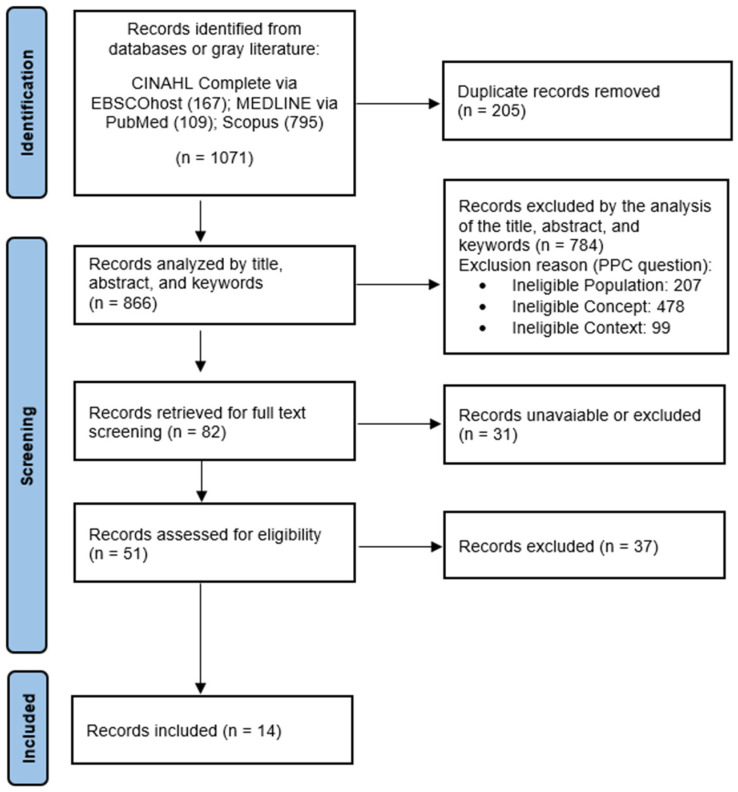
Study identification and inclusion process—PRISMA-ScR diagram flow (adapted).

As shown in Figure 1, the search identified 1071 potentially relevant studies coming from the previously defined databases, with no studies being identified from the gray literature search. Of these, 205 were excluded for being duplicates, and, among the remaining 866 studies, 784 were removed after analyzing the title and abstract, and 37 were excluded for not meeting the inclusion criteria after reading the full text. In the end, 14 studies were included in this review.

Five studies are from Spain, and two are from South Africa. The countries of origin with one study are Saudi Arabia, Brazil, Lithuania, Taiwan, India, United Arab Emirates, and China. The publication of the included studies occurred between 2002 and 2021. There has been an intensification of studies in more recent years, with 2018 being the year having the highest number of included studies, with three studies. Ten studies are in the English language, three in Spanish, and one in Portuguese. Regarding the methodological design, eight studies were correlational, three were descriptive studies, one was methodological, one was a cohort study, and one was an integrative literature review.

The number of nurses that represents the samples of the included studies totals 4304 nurses, whose main activity focuses on caring for families, with the smallest sample comprising 64 nurses and the largest comprising 969 nurses.

The fourteen studies included refer to ten self-report assessment instruments. Nine of these instruments analyze workload as a component or dimension of a broader instrument whose main objective was to evaluate another conceptual construct, such as stress, moral stress, burnout, quality of professional life, or professional satisfaction, presenting heterogeneous structures.

Only two studies refer to the same instrument (quantitative workload inventory), which focuses exclusively on workload, and even in these cases, as an integrating part of a multidimensional questionnaire whose objective was to assess family nurses’ burnout.

The number of items in the included instruments varies between 5 and 12, and the most common response format is the five-point Likert scale.

No studies comparing instruments were found.

The different studies analyzed are summarized in Table 2.

## 4. Discussion

The studies included in this review allow us to state that there is still a gap in evidence regarding family nurses’ workload assessment [1,17,22,23,28,29]. We need to be cautious when using conclusions from previous studies that analyze workload as a phenomenon with similar characteristics in family nurse care and hospital care, as the nature of care provided by family nurses is different from that provided in hospitals. [30]. Metrics such as the time spent while performing care or the amount of care activities carried out by family nurses seem to be referred to often in the included studies, but these aspects tend to neglect the complex and broad nature of the family nurses’ scope of practice [8].

This stands as a major concern that several authors have been highlighting, referring to the importance of a more comprehensive approach to the assessment of family nurses’ workload that includes aspects like work-life balance, compassion fatigue, or even nurses’ resilience as variables with an impact on a family nurse’s workload [8,9,10].

The small number of studies included in this scoping review, when compared with other similar reviews for other care settings [4,31,32], also seems to clearly demonstrate the lack of specific instruments for evaluating a family nurse’s workload.

This difficulty is due, in part, to the great latitude regarding the family nurses’ scope of action. The analyzed studies reported, in a heterogeneous way, a diverse range of family nurses’ professional skills, roles, and competencies. This means that the skills, professional roles, and competencies of Portuguese family nurses, that we considered as a basis for formulating our research questions, under the Portuguese legal framework [33,34,35], may be configured differently in other countries and health systems [8], which can be seen as a limitation to the present review.

Several studies that were included reflect this aspect in the instruments used [1,20,22,24,28], making clear the wide range of skills, roles, competencies, and care performed by family nurses, introducing heterogeneity in the items that make up the workload assessment instruments.

The differences pointed out by the included studies in this matter go beyond terminology, as they tend to reflect the philosophy present in each health care system, for primary health care nurses and the way they view family care. These differences also extend to the operationalization of health systems and the organization and planning of how care reaches populations, families, or individuals in primary health care [8].

In Portugal, family nurses, integrated into multidisciplinary health teams, assume responsibility for providing global nursing care to a limited group of families, in all the life-cycle health processes, in the various community settings, and each family nurse is entrusted to care for 300 to 400 families [33,34,35].

The realities are, therefore, unique. However, in this review, we seek the rigor of reporting studies that are aligned with the reality of nursing care provided to families in Portugal and that allow us to identify a great professional affinity in terms of autonomy, responsibilities, and professional skills, with the role of family nurses in Portugal.

This review also identified, as reflected in the instruments that evaluate the family nurses’ perceived workload, the polysemy, and broadness of this concept, confirming its wide range of coverage, which seems to point to the need for a more globalizing and integrating perspective of the different aspects that are conceptually close and relevant to the workload construct. The operative definitions contained in the instruments are not consensual and, despite not being antagonistic, they refer to an overlap of premises or aspects that characterize them. The reviewed studies include a wide range of concepts that, although conceptually independent, indirectly make up the overall perceived workload [1,17,19,20,23,26,28]. An example of this is instruments that point out factors such as work pressure [1,17,19,21,28], the pace of work [20,23], the time available to provide care [1,17,19,21,28], the impact of work on family life [25,26,27], staffing/professional ratios [1,17,19,21], carrying out administrative or non-care-related tasks [17], physical, cognitive and emotional effort [26] or the availability of resources [1,29]. Some of these aspects are more prevalent in the revised instruments; however, it is premature to conclude that these are more significant than others for evaluating a family nurse’s workload.

This conceptual broadness seems to also dictate the difficulty of identifying specific instruments for evaluating workload in this care context.

The studies included in the present review mainly aimed to evaluate family nurses’ workload in two different ways: (1) with their own instrument (integrating a multidimensional questionnaire) or (2) as a component of another instrument (in the form of a dimension), in a specific workload sub-scale.

The review carried out also allows us to list, as result of the number of correlational studies included [17,19,21,22,23,26,28,29], the relationship between family nurses’ perception of workload and its main consequences. Burnout stands out [21], and professional dissatisfaction [19,26,28], illness and professional absenteeism [17,22], and the intention to leave the current workplace or even nursing [23,28], were the major workload consequences depicted in studies included in our review. The included integrative review [1] also demonstrates the relationship between workload and the quality and safety of care provided by family nurses.

It’s important to note that in certain studies [17,19,20], indirect care (non-patient-related activities) significantly influences the perception of workload, but it is challenging to assess due to its subjective nature. These activities are often referred to as the hidden or submerged face of workload. [14,36]. Among them, documentation of care [17] and carrying out administrative tasks [19,20] stand out.

In the instruments analyzed, a heterogeneous and diversified structure is evident, which, despite not being the objective of this study, makes the comparison of studies unfeasible. We can therefore state that this fact attests to the absence of a standard instrument to assess the workload of family nurses. Even so, each instrument has advantages and disadvantages; therefore, it is important to highlight that, for the appropriate choice of an instrument, the focus must be on the assessment of the overall workload.

It also becomes evident, with the findings of this review, that the studies included are not based on a clearly defined theoretical framework. Only one study [24] refers to Karasek’s Demand and Control Model as the conceptual framework used.

There are several theories and models that explain workload, many of them dedicated to health settings [37,38]. Their focus of analysis varies between ergonomics and the impact of the volume of work on the physical and psychological conditions of workers, especially those more exposed to risk factors associated with the provision of care [39]; the mental load associated with the complexity of tasks carried out [40]; and the relationship between the demand for care and the availability of resources [41]. It should be noted that there is currently a tendency towards an integrative or globalizing perspective of the different dimensions that can shape workload perception, including the following in the same theoretical perspective: supply and demand, the cognitive complexity associated with the provision of care, the physical and emotional burden, the availability of resources and, among these, the time to perform care, as a broad, comprehensive and global perspective of the aspects that embody nurses’ workload assessment.

We consider that the set of studies included in the present review allows us to conclude that the multiplicity of professional skills, roles, and competencies of family nurses, exercised throughout the families’ and individuals’ life cycle, associated with the conceptual diversity of workload assessment, and the absence of a clearly defined theoretical framework, makes it difficult to identify unique or consensual instruments that allow for family nurse workload assessments. It should be noted that these findings are congruent with existing evidence in this area [30] and are in line with authors [41] who stated that the complexity of nursing care and technological advances highlight the need to review and update workload quantification systems.

As limitations to the present review, we can state that the literature on workload is complex, with a wide variety of constructs and operationalizations to represent it, often with little coherence in the use of terminology. This means that there may be terms that belong to the workload domains that we did not include in the search. As a result, some instruments may have been overlooked by the procedures we followed. Likewise, our results may also be influenced by a certain degree of reporting bias, as researchers may be less willing to publish unfavorable results in terms of the psychometric properties of an instrument.

Another limitation that we can point out refers to the lack of existing literature reviews focusing on the professional settings of family nurses. The initial research in this area is preliminary, so our review is based on a limited and scarce initial pool of studies, particularly when compared to the ones produced in the hospital setting, that could have affected the depth of our analysis. It is therefore evident that this issue is still little explored, reflecting a gap to be filled with future research.

## 5. Conclusions

This scoping review intended to provide a valuable foundation for understanding the current landscape of family nurses’ workload assessment tools. Through a comprehensive approach and methodological rigor, we intended to contribute, as a preliminary exercise, to the identification of gaps in the literature that encourage carrying out future primary studies and optimizing research designs and methodologies, that justify the formulation of new questions.

Workload assessment is an important factor that can contribute to improving the quality of nursing care provided. This review focused on existing instruments in the literature, which allows for the assessment of family nurses’ workload, establishing a starting point for the analysis and systematization of the main evidence available in this area.

Our review demonstrates that, in addition to the heterogeneity and the small number of instruments that quantitatively assess the workload of family nurses, there is no broad consensus on which instrument is best, making comparison of the results unfeasible.

For that reason, this review intends to establish a guide, as it highlights the importance of deepening the knowledge on this issue to obtain reliable, broad, and integrative instruments for the overall care provided by family nurses, which allows for an inclusive and comprehensive portrayal of the care provided, clarifying which dimensions of workload are most relevant to be measured by nurses in the practice scenario in question.

The scarcity of studies leads to a lack of consensus in this area; the limited focus on specific but increasingly important areas, such as emotional aspects, as well as insufficient exploration of primary care-specific metrics, highlight the need for targeted research and refinement in this field. Addressing this in future studies could enhance the accuracy and applicability of workload assessments for family nurses.

The authors of this study affirm that they do not have any connections with funding institutions or other entities that may benefit from their results and could create potential conflicts of interest. 

## Figures and Tables

**Table 1:** Example of search on Medline (via PubMed) on 23 March 2022.

Example of Search String	Hits
(((“workload”[Title/Abstract])AND(“assessment”[Title/Abstract]OR“evaluation”[Title/Abstract]OR“instrument”[Title/Abstract]OR “scale”[Title/Abstract]OR“measurement”[Title/Abstract]))AND(“nursing”[Title/Abstract]OR“nurse”[Title/Abstract]OR“nurses”[Title/Abstract]))AND(“primary care”[Title/Abstract]OR“primary health care”[Title/Abstract])	109

**Table 2:** Included studies and relevant results.

Title (Authors/Year)	Instrument	Dimensions (Number of Items)	Workload Sub-Scale (Number of Items)	Internal Consistency (Cronbach’s Alpha)	
				Instrument	Sub-Scale
Alenezi et al. (2018) [17]—Saudi Arabia	Nursing stress scale	7 (34 items)	Workload (6 items)	0.94	--
Barth et al. (2018) [18]—Brazil	Moral stress scale	6 (46 items)	Overload (5 items)	0.98	0.88
Bester & Engelbrecht (2009) [19]—South Africa	Multidimensional instrument built by the researchers	no dimensions identified (5 items)	Quantitative Workload Inventory (5 items)	--	0.85
Cortés-Rubio et al. (2003) [20]—Spain	PQL-35 (Spanish version)	3 (35 items)	Workload (12 items)	--	0.75–0.86
Engelbrecht et al. (2008) [21]—South Africa	Multidimensional instrument built by the researchers	no dimensions identified (5 items)	Quantitative Workload Inventory (5 items)	--	0.85
Galdikien et al. (2014) [22]—Lithuania	Expanded nursing stress scale	9 (55 items)	Workload (8 items)	0.92	0.85
Lee & Wang (2002) [23]—Taiwan	Stressors scale	6 (40 items)	Workload (6 items)	0.96	0.83
Martin-Fernandez et al. (2007) [24]—Spain	PQL-35 (Spanish version)	3 (35 items)	Workload (12 items)	--	0.75–0.86
Panadero & Madroño (2012) [25]—Spain	Font-roja questionnaire	9 (26 items)	Work Pressure (3 items)	--	0.79
Pérez-Francisco et al. (2020) [1]—Spain	Work conditions assessment scale	3 (31 items)	Work Conditions (11 items)	--	--
Purohit & Banopadhyay (2021) [26]—India	Measure of job satisfaction	7 (40 items)	Satisfaction with Workload (9 items)	0.95	0.85
Suleiman & Adam (2020) [27]—United Arab Emirates	Measure of job satisfaction	7 (40 items)	Satisfaction with Workload (9 items)	0.95	0.85
Tao et al. (2018) [28]—China	Work stress scale (WSS) Job satisfaction scale (JSS)	WSS: 5 (40 items) JSS: 8 (35 items)	Workload (WSS: 5 items) (JSS: 5 items)	WSS—0.98; JSS—0.86	--
Castro et al. (2015) [29]—Spain	PQL-35 (Spanish version)	3 (35 items)	Workload (12 items)	0.80–0.85	--

## Data Availability

For data supporting reported results, please contact the authors of this review.

## Public Involvement Statement

There was no public involvement in any aspect of this research.

## Guidelines and Standards Statement

This manuscript was drafted according to the recommendations of The Joanna Briggs Institute for scoping review research.
